# Thermophysiological and Perceptual Responses of Amateur Healthcare Workers: Impacts of Ambient Condition, Inner-Garment Insulation and Personal Cooling Strategy

**DOI:** 10.3390/ijerph20010612

**Published:** 2022-12-29

**Authors:** Yingying Zhao, Meng Su, Xin Meng, Jiying Liu, Faming Wang

**Affiliations:** 1 School of Thermal Engineering, Shandong Jianzhu University, Jinan 250101, China; 2 Department of Biosystems, Katholieke Universiteit Leuven, 3001 Leuven, Belgium

**Keywords:** COVID-19, healthcare workers, personal protective equipment, thermal comfort, personal cooling

## Abstract

While personal protective equipment (PPE) protects healthcare workers from viruses, it also increases the risk of heat stress. In this study, the effects of environmental heat stress, the insulation of the PPE inner-garment layer, and the personal cooling strategy on the physiological and perceptual responses of PPE-clad young college students were evaluated. Three levels of wet bulb globe temperatures (WBGT = 15 °C, 28 °C, and 32 °C) and two types of inner garments (0.37 clo and 0.75 clo) were chosen for this study. In an uncompensable heat stress environment (WBGT = 32 °C), the effects of two commercially available personal cooling systems, including a ventilation cooling system (VCS) and an ice pack cooling system (ICS) on the heat strain mitigation of PPE-clad participants were also assessed. At WBGT = 15 °C with 0.75 clo inner garments, mean skin temperatures were stabilized at 31.2 °C, *H*_skin_ was 60–65%, and HR was about 75.5 bpm, indicating that the working scenario was on the cooler side. At WBGT = 28 °C, *T*_skin_ plateaued at approximately 34.7 °C, and the participants reported “hot” thermal sensations. The insulation reduction in inner garments from 0.75 clo to 0.37 clo did not significantly improve the physiological thermal comfort of the participants. At WBGT = 32 °C, *T*_skin_ was maintained at 35.2–35.7 °C, *H*_skin_ was nearly 90% RH, *T*_core_ exceeded 37.1 °C, and the mean HR was 91.9 bpm. These conditions indicated that such a working scenario was uncompensable, and personal cooling to mitigate heat stress was required. Relative to that in NCS (no cooling), the mean skin temperatures in ICS and VCS were reduced by 0.61 °C and 0.22 °C, respectively, and the heart rates were decreased by 10.7 and 8.5 bpm, respectively. Perceptual responses in ICS and VCS improved significantly throughout the entire field trials, with VCS outperforming ICS in the individual cooling effect.

## 1. Introduction

Since the outbreak of the novel coronavirus in 2019, the virus has spread globally [1,2] and evolved into various variants [3,4], posing a significant challenge to the national healthcare system [5]. Personal protective equipment (PPE) for healthcare workers is unquestionably an important component in the prevention and control of infection during pandemics [6,7]. PPE typically consists of protective clothing, goggles, shoe covers, and respiratory protection equipment [8]. Disposable medical protective clothing is typically made of “PP non-woven + PE laminate” composite material. This material has a dense structure that protects healthcare workers from viral infection but causes the gowns to exhibit poor air permeability and thermal comfort [9]. Prolonged use of PPE can cause dizziness, discomfort, and headache [10,11]. Some reports have indicated that PPE-clad healthcare workers soak and sweat profusely while performing nucleic acid detection tasks in the hot summer [12]. Some workers have even suffered from dehydration, heat cramp, heat stroke, and other symptoms that can be fatal in severe cases. Thus, recognition of the potential dangers of heat stress to the health and safety of healthcare workers is critical [13], as is the improvement of the thermal comfort of medical personnel without compromising PPE protection performance [14,15].

Numerous studies have studied the thermal comfort of PPE-clad healthcare workers [16,17]. Loibner et al. [18] investigated the physical limitations caused by PPE-induced restrictions. They concluded that too many layers of gloves lead to reduced flexibility and that heat stress and fluid loss are the main limiting factors in high-temperature environments. Grélot et al. [19] examined the level of heat stress experienced by healthcare workers while wearing PPE during the Ebola outbreak. According to this study, the average time spent by healthcare workers treating patients with Ebola virus disease under hot conditions in West Africa was 65.7 min, during which they were not at risk of heat stress. However, approximately 4 of every 25 healthcare workers had core temperatures that exceeded the recommended working limit of 38.5 °C. Using experiments and surveys, Mao et al. [20] determined the thermal comfort and physiological indices of participants wearing and not wearing PPE when collecting nucleic acid samples outdoors. The comfort temperature zones of participants before wearing PPE were found to be 17.4–21.5 °C; meanwhile, participants wearing PPE required a cooler working environment to maintain thermal comfort. In a previous study [21], field experiments were used to analyze and evaluate the thermal comfort of participants wearing medical protective clothing for nucleic acid detection. The findings emphasized the need for measures to mitigate heat stress in nucleic acid sampling personnel working in high-temperature environments.

Personal cooling measures can effectively alleviate heat stress response in PPE-clad workers [22,23,24]. Wang et al. [25] evaluated the effectiveness of using cooling suits to reduce heat stress in mascot actors working in a hot and humid environment. Both the hybrid cooling suit and the water-cooled suit considerably reduced heat stress in mascot wearers. Su et al. [26] developed a type of medical-use protective clothing (MUPC) with a portable cooling device. A simulation method was employed to examine the environmental, physiological, and subjective evaluation parameters of the subjects in MUPC. The results showed that a cooling airflow rate of 50 L/min with a supply temperature of 18–20 °C could ensure a thermally comfortable microclimate in MUPC. Wu et al. [27] studied the cooling capacity of personnel wearing ventilation vests (VCVs) while performing manual labor in environments at different temperatures. They concluded that the torso skin temperature of personnel wearing VCVs was reduced: total torso heat loss was increased by 169–237% and the cooling power of VCVs ranged from 79.5 W to 97.6 W.

Currently, numerous studies have been conducted on human thermal comfort while wearing medical PPE, but most were performed in the laboratory. More PCR testing sites are found in outdoor environments, where the outdoor climate is changeable and air-conditioned rooms and power supplies to the cooling system are not present. Relatively few studies have been reported on outdoor environment testing and portable cooling. Therefore, in the present study, the effects of environmental heat stress, inner-clothing layer insulation, and personal cooling strategy on the physiological and perceptual responses of COVID-19 PPE-clad young college students were evaluated. This study aimed to provide a reference for improving the thermal comfort of healthcare workers while wearing COVID-19 PPE during field PCR testing.

## 2. Methods

### 2.1. Field Study Conditions and Instruments

**Figure 1:**
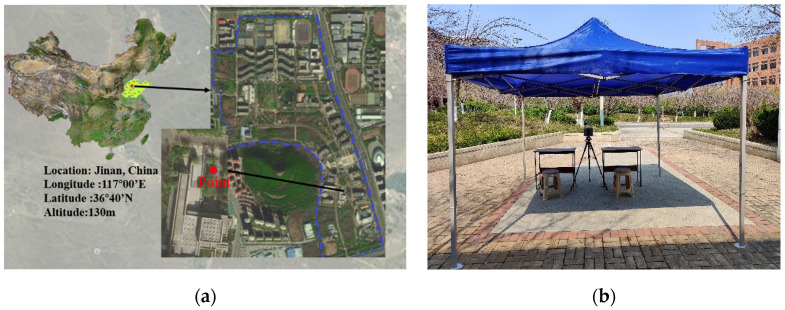
Pictures of the experiment site. (**a**) Location of the testing site. (**b**) Layout of the experimental site.

The experiment was conducted on the campus of Shandong Jianzhu University (Figure 1a). Temporary tents were erected in an open area with good ventilation and lighting (Figure 1b). Each tent measured 3 m in length, 3 m in width, and 1.9 m in height to simulate the working environment of healthcare workers. From March to August, 2 participants at a time were tested for 2 h.

**Figure 2:**
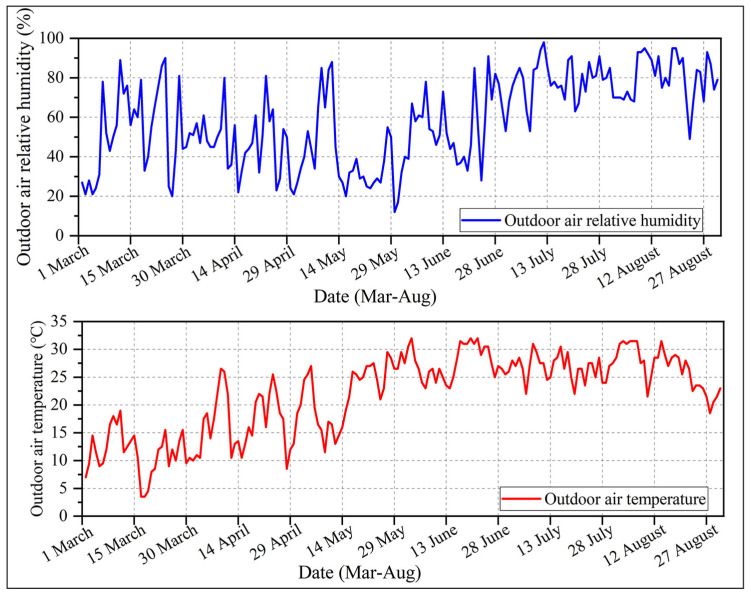
Outdoor weather data acquired during the experimental period from 1 March to 31 August 2022.

Figure 2 depicts the weather conditions during the testing period. The meteorological data were acquired from Station 54823 in Jinan, Shandong Province [28]. Environmental parameters were measured using the JT-IAQ thermal environment and comfort monitor during the experiment, and data were recorded at intervals of 1 min. The equipment was set up between two floor tables, with the center 1.1 m above the ground. The oral temperature was measured using the YCT-2 thermometer. The temperature-sensing probe was placed under the tongue near the artery, and a data set was collected every 15 min, with each set of data containing three consecutive measurements. To reduce measurement errors, the average value was used. With an iButton temperature and humidity recorder, the skin temperature was measured at eight points: the forehead, the right scapula, the left upper chest, the right upper arm, the left lower arm, the left hand, the right anterior thigh, and the left calf. The device can measure the relative humidity of the skin surface and the skin temperature. The iButton was taped down with a medical pressure-sensitive tape, and data were collected at intervals of 1 min. The heart rate of the participant was measured with a Polar heart rate strip, and the data were recorded every 1 s. Table 1 lists the instruments used in the field study.

### 2.2. Experimental Environment and Instruments

People from different age brackets were difficult to invite for testing on campus because of epidemic control measures. Thus, the majority of the participants in this experiment were students—a total of 23 participants without bad habits, including 9 females and 14 males. Table 2 lists all participants who were senior students and had lived in Jinan for more than 3 y. Before the experiment, all participants were informed of the purpose of the experiment and the precautions. Perceptual responses, including thermal sensation vote (TSV), humid sensation vote (HSV), thermal comfort vote (TCV), Borg’s rating of perceived exertion (RPE), and thermal expectation vote (TEV) were included in the surveys (Table 3).

### 2.3. Experimental PPE and Ensembles

The outer clothing used for the experiments in the basic trial is a full PPE set, including medical protective clothing, goggles, disposable PVC gloves, an isolation mask, and disposable isolation shoe covers. The inner garment consists of two sets of clothing with significant differences in thermal resistance. The thermal resistance of the inner clothing layer is calculated using Equation (1) by comparing it to the thermal resistance table of a single garment. The thermal resistance values of the inner garments were 0.75 and 0.37 clo, respectively. In Dress1, the inner garments consisted of a long-sleeve top and trousers, which belong to the double-sided brushed fabric. In Dress2, the inner garments consisted of a T-shirt and shorts. The T-shirt was made of modal fabric, and the shorts were made of polyester. In the cooling trials, two types of personal cooling suits were used—one based on a reusable ice pack cooling system (ICS) and another based on a ventilation cooling system (VCS) with an active air supply device—to consider the mobility of the outdoor nucleic acid detection point and the portability of the healthcare workers. The 0.37 clo inner garments were chosen for the cooling trials. An ice pack cooling vest with a length of 53 cm and a chest circumference of 92 cm was added to the ICS system. One vest can hold up to 12 ice packs with a capacity of 200 mL. The ice packs were prefrozen in the refrigerator and transported to the testing site in an incubator. During the experiment, 12 ice packs were placed on each vest, and the total weight of the vest was 2.57 kg. The VCS system used the same material as the regular protective clothing, but two air inlets with a diameter of 2 cm had been added in the chest position, and a small ventilator with a maximum ventilation capacity of 9 L/S had been added to the back. The outside air was filtered via 3M^TM^ 2097 P100 high-efficiency cotton filter sheets before it was delivered to the air inlet at the chest via the ventilation tube in the back. The 2097 P100 cotton filter sheets could achieve at least 99.97% particulate filtration efficiency, effectively protecting against viruses. The fan was powered by an 8000 mAh lithium-ion battery that could last for 8 h. Table 4 lists the detailed experimental dressing.

### 2.4. Experimental Setup

By calculating the mean WBGT index of each participant during the experiment and combining the most typical meteorological parameters in different months in Jinan, the most representative WBGT values in March–April, May–June, and July–August were calculated and screened out to be 15 ± 0.5 °C, 28 ± 0.5 °C and 32 ± 0.5 °C, respectively. The data with large deviations in environmental parameters and the data with extreme physiological parameters and survey parameters were eliminated. In the basic trials, a thicker fabric with *I*_cl_ = 0.75 clo was selected for WBGT = 15 °C tests. The temperature during the day varied largely from that during the May–June period. Two types of inner garments (*I*_cl_ = 0.75 and 0.37 clo) were selected for the experiment. The 0.37 clo inner garments were chosen for the experiment at 32 °C WBGT. Thus, six different working scenarios are presented in this study. The cooling trial was an improvement experiment based on the basic experiment and its results. The effects of ICS and VCS cooling measures on the physiological and perceptual responses were evaluated at WBGT = 32 °C and *I*_cl_ = 0.37 clo. The experimental parameter for basic protective clothing without a cooling system in the same environment was used as the control parameter; that is, NCS.

**Figure 3:**
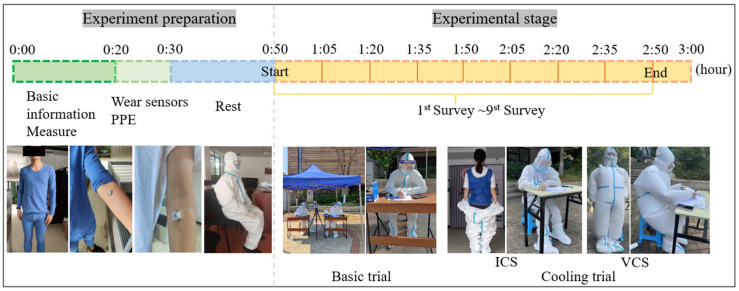
Schematic showing details of experimental procedures.

Figure 3 presents the experimental procedure. A simple changing room was constructed in an office near the experimental site. Each group of experiments included two participants who wore heart rate belts, pasted skin temperature and humidity sensors, dressed in the changing room, and walked to the experimental site. Participants were required to sit still and adjust themselves upon arrival at the site to ensure that no serious discomfort was experienced before the experiment began. The entire test lasted for 2 h, with questionnaires filled out and oral temperatures taken every 15 min.

### 2.5. Evaluation Indices

The thermal resistance and moisture resistance of clothing are two important indicators of its heat and moisture transfer performance. Thermal resistance can also be unified in human thermal comfort experiments by unifying clothing. To quantify clothing thermal resistance, ASHRAE standard clothing thermal resistance is used as a fixed value. The thermal resistance of the entire set of clothing (*I*_cl_) is determined by superimposing the thermal resistance of a single piece of clothing (*I*_cl,i_) [29]:

(1) $$I_{\mathrm{cl}}=0.835\sum_{i}I_{\mathrm{cl},i}+0.161$$

The wet bulb globe temperature (WBGT) index is the most commonly used index for evaluating environmental thermal stress. It is mainly used to determine safe exposure limits in hot and humid environments. This index is calculated using three parameters: black globe temperature (*t*_g_), which reflects the solar radiation; wet bulb temperature (*t*_w_); and dry bulb temperature (*t*_a_). The calculation equation of WBGT for outdoor use with solar radiation is as follows [30]:

(2) $$WBGT=0.7t_{w}+0.2t_{g}+0.1t_{a}$$

In the current study, the mean skin temperature ($T_{\mathrm{skin}}$) was calculated using eight body locations to obtain a reliable mean skin temperature (Equation (3)), and the mean skin humidity is calculated using the weight coefficient of the mean skin temperature in Equation (4) [25].

(3) $$T_{\mathrm{skin}}=0.07t_{\mathrm{forehead}}+0.175t_{\mathrm{right}\;\mathrm{scapular}}+0.175t_{\mathrm{left}\;\mathrm{upper}\;\mathrm{chest}}+0.07t_{\mathrm{right}\;\mathrm{upper}\;\mathrm{arm}}+0.07t_{\mathrm{left}\;\mathrm{lower}\;\mathrm{arm}}+0.05t_{\mathrm{left}\;\mathrm{hand}}+0.19t_{\mathrm{right}\;\mathrm{anterior}\;\mathrm{thigh}}+0.2t_{\mathrm{left}\;\mathrm{calf}}$$

(4) $$H_{\mathrm{skin}}=0.07H_{\mathrm{forehead}}+0.175H_{\mathrm{right}\;\mathrm{scapular}}+0.175H_{\mathrm{left}\;\mathrm{upper}\;\mathrm{chest}}+0.07H_{\mathrm{right}\;\mathrm{upper}\;\mathrm{arm}}+0.07H_{\mathrm{left}\;\mathrm{lower}\;\mathrm{arm}}+0.05H_{\mathrm{left}\;\mathrm{hand}}+0.19H_{\mathrm{right}\;\mathrm{anterior}\;\mathrm{thigh}}+0.2H_{\mathrm{left}\;\mathrm{calf}}$$

### 2.6. Statistical Analysis

Discrete values during the experiment were used to analyze mean skin temperature and humidity, heart rate, core temperature, TSV, TCV, HSV, and RPE every 5 min. The software IBM SPSS Statistics 25 was used to conduct a one-way repeated measures ANOVA to evaluate the physiological parameters and perceptual responses across time (every 5 min for physiological variables or every 15 min for perceptual variables) under various working conditions to examine statistical differences between trial conditions. If a significant effect was observed, a Bonferroni-corrected paired sample t-test was performed to analyze the differences in physiological parameters and subjective questionnaire parameters under the different working conditions. Differences were considered statistically significant at the *p* < 0.05 level. All values are expressed as mean ± standard deviation.

## 3. Results

### 3.1. Physiological Responses in Basic Trials

#### 3.1.1. Mean and Local Skin Temperatures

**Figure 4:**
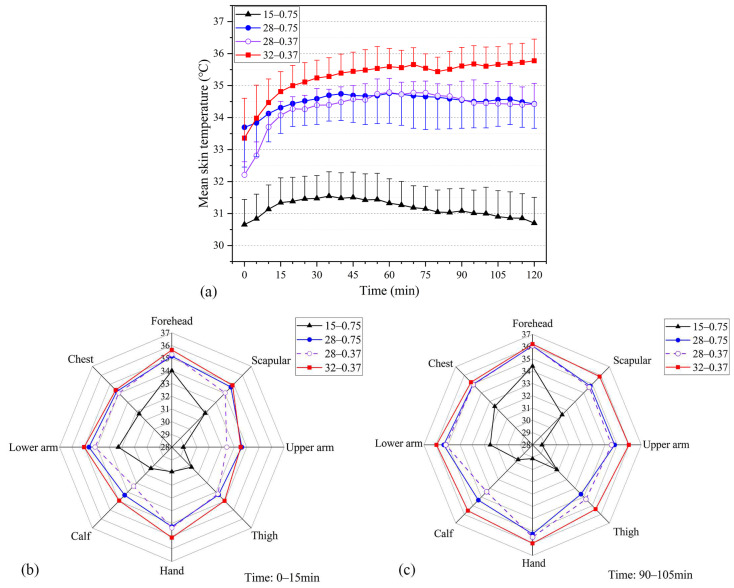
Skin temperature responses under four testing scenarios (i.e., 15 °C–0.75 clo, 28 °C–0.75 clo, 28 °C–0.37 clo, 32 °C–0.37 clo; testing scenarios in the figures are presented as A–B, where A represents the WBGT value and B represents the insulation of the inner garments used in the testing). (**a**) Mean skin temperatures. (**b**) Local mean skin temperatures at 0–15 min. (**c**) Local mean skin temperatures at 90–105 min.

Figure 4 depicts the skin temperature responses under the four working conditions. The 0.75 clo inner garments were used at WBGTs of 15 °C and 28 °C, whereas the 0.37 clo inner garments were used at WBGTs of 28 °C and 32 °C. The mean skin temperature increased from 0 to 15 min (Figure 4a). The *T*_skin_ value of the participants at WBGT = 15 °C was significantly lower than the measured values at WBGT = 28 °C and 32 °C, with a mean temperature of 31.16 °C. Over time, the human body feels cold and desires warmth, hence the slow decline in mean skin temperature. At WBGT = 28 °C, the mean skin temperature when 0.75 clo inner garments were worn was slightly higher than that when 0.37 clo inner garments were worn. The sweat of the participants had partially evaporated, removing some of the heat from the surface layer of the body; consequently, the human skin temperature slightly tended to decrease after increasing to a certain level. *T*_skin_ at WBGT = 32 °C was significantly higher than *T*_skin_ at WBGT = 28 °C throughout the period, with a difference of about 1 °C. *T*_skin_ reached 35.3 °C at WBGT = 32 °C.

The variability of skin temperature in different parts of the human body was indicated by the local skin temperature, which is also an important reference value for improving human thermal comfort. Figure 4b,c compare the mean local skin temperature of participants under each working condition at 0–15 and 90–105 min, respectively. The head temperature was at a relatively high temperature under all working conditions. The measured skin temperature at WBGT = 15 °C was significantly lower than that under other working conditions, and the low-temperature environment significantly affected the skin temperature of more prominent or slender parts, such as the scapula, arm, hand, leg, and so on. At 0–15 min, the skin temperature of the trunk (chest, scapula, and upper arm) at WBGT = 28 °C and *I*_cl_ = 0.75 clo was close to the measured value at WBGT = 32 °C and *I*_cl_ = 0.37 clo and higher than that at WBGT = 28 and *I*_cl_ = 0.37 clo. At 90–105 min, the difference in the skin temperature of the trunk (chest, scapula, and upper arm) was not obvious under a different clothing thermal resistance at WBGT = 28 °C.

#### 3.1.2. Skin Relative Humidity

**Figure 5:**
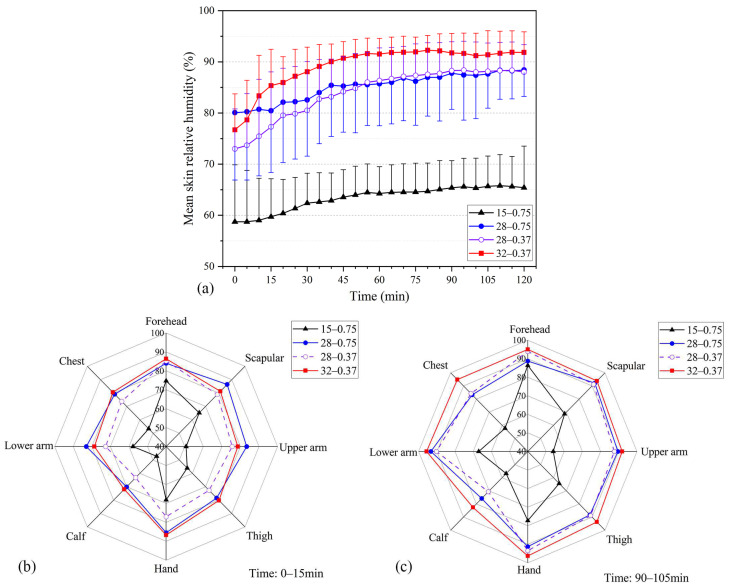
Skin relative humidity under the four testing scenarios (i.e., 15 °C–0.75 clo, 28 °C–0.75 clo, 28 °C–0.37 clo, 32 °C–0.37 clo; testing scenarios in the figures are presented as A–B, where A represents the WBGT value and B represents the insulation of the inner garments used in the testing). (**a**) Mean skin relative humidity. (**b**) Local mean skin relative humidity at 0–15 min; (**c**) Local mean skin relative humidity at 90–105 min.

Figure 5 presents the relative humidity change trend of human skin under various working conditions. *H*_skin_ at WBGT = 32 °C was the highest value, reaching 90% RH after 40 min and remaining at that level (Figure 5a). *H*_skin_ at WBGT = 28 °C was the next highest value, rising to nearly 85% RH after 40 min and gradually approaching 90% RH. Overall, the *H*_skin_ value of the participants at WBGT = 15 °C was lower, rising from 60% RH to 65% RH during the experiment. *H*_skin_ at *I*_cl_ = 0.75 clo was higher than *I*_cl_ = 0.37 clo at the start of the experiment, and the two gradually converged after 40 min as the experiment progressed. This occurrence indicates that the two dressing patterns affected *H*_skin_, but reducing the thermal resistance of the inner garments did not reduce sweating in the participants.

Figure 5b,c compare the mean local skin relative humidity of participants under each working condition at 0–15 and 90–105 min, respectively. At 0–15 min, skin humidity in every part of the skin of participants in *I*_cl_ = 0.75 clo was higher than that of participants in *I*_cl_ = 0.37 clo at WBGT = 28 °C. *H*_skin_ in the scapula and arm was even higher than *H*_skin_ at WBGT = 32. This difference was mainly caused by wearing thick inner garments in a hot environment. At 90–105 min, the relative humidity of the trunk (chest, scapula, and upper arm) was gradually approaching. Local skin relative humidity at WBGT = 32 °C was higher than the measured value at WBGT = 28 °C. This difference indicates that as the experiment progressed, the effect of clothing thermal resistance on skin humidity gradually decreased, and the effect of environmental parameters on skin humidity gradually increased because of the participants being in a hot state for an extended time.

#### 3.1.3. Core Temperature and Heart Rate

**Figure 6:**
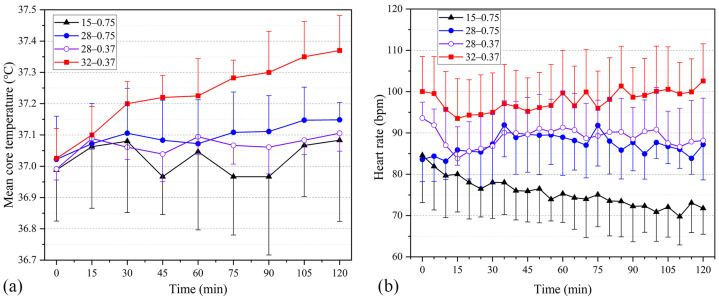
Temporal variation of *T*_core_ and HR under four different working conditions (i.e., 15 °C–0.75 clo, 28 °C–0.75 clo, 28 °C–0.37 clo, 32 °C–0.37 clo; testing scenarios in the figures are presented as A–B, where A represents the WBGT value and B represents the insulation of the inner garments used in the testing). (**a**) Mean core temperature. (**b**) Heart rate.

Figure 6a presents the temporal variations in core temperature under the four testing scenarios. The core temperature at WBGT = 15 °C more widely fluctuated than the remaining three testing scenarios. Core temperatures at WBGT = 32 °C were significantly higher by about 0.15 °C than the reported values at WBGT = 28 °C. The core temperatures with 0.75 clo inner garments were slightly higher than those with 0.37 clo inner garments at WBGT = 28 °C. The core temperature did not exceed 37.5 °C at any point during the experiment.

Figure 6b displays the time course heart rate (HR) responses under the four working conditions. Heart rate was influenced by interindividual variability, resulting in relatively large standard deviation values of HR under each working condition. The mean heart rates were 75.5 and 87.2 bpm, respectively, with the 0.75 clo inner garments at WBGTs of 15 °C and 28 °C. By contrast, HRs were 89.1 and 91.9 bpm, respectively, with the 0.37 clo inner garments at WBGTs of 28 °C and 32 °C. The mean HR values increased by 11.7, 13.6, and 16.6 bpm, respectively, relative to those at WBGT = 15 °C, indicating that the higher the heat stress, the greater the tendency for HR to rise. No significant difference in HR measurements was found between the two types of inner garments at WBGT = 28 °C.

### 3.2. Perceptual Responses in Basic Trials

**Figure 7:**
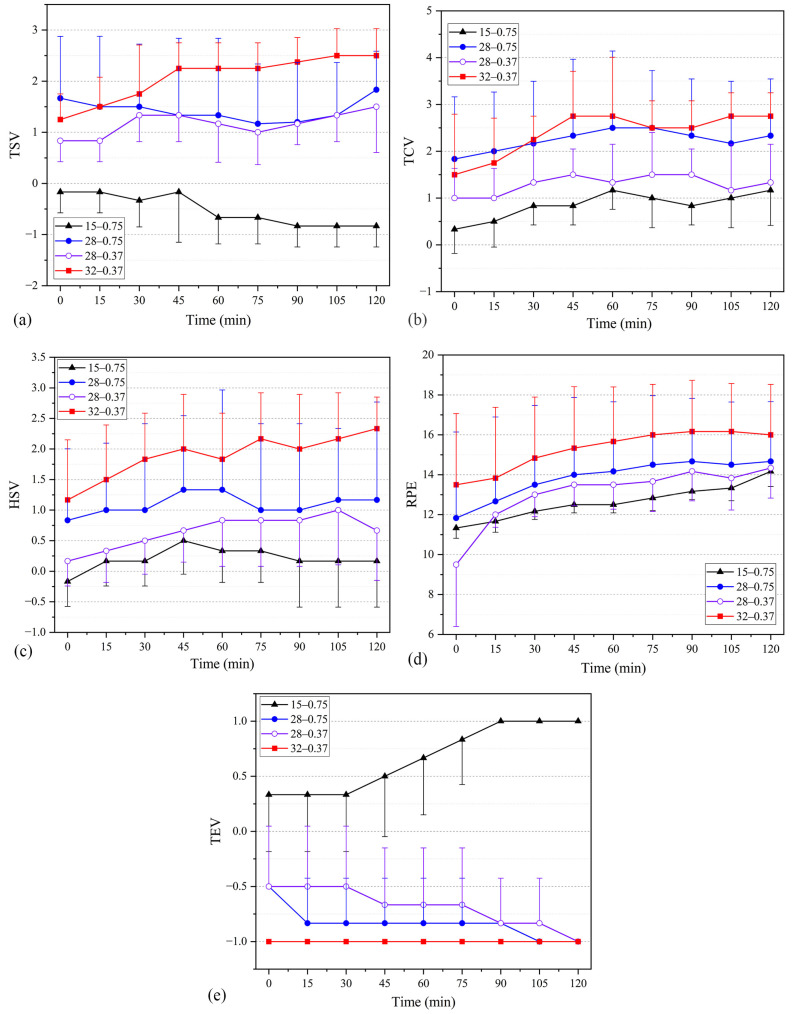
Temporal variations of thermal sensation votes, thermal comfort votes, humidity sensation votes, and ratings of perceived exertion under four different working conditions (i.e., 15 °C–0.75 clo, 28 °C–0.75 clo, 28 °C–0.37 clo, and 32 °C–0.37 clo; testing scenarios in the figures are presented as A–B, where A represents the WBGT value, and B represents the insulation of the inner garments used in the testing). (**a**) Thermal sensation votes. (**b**) Thermal comfort votes. (**c**) Humid sensation votes. (**d**) Borg’s rating of perceived exertion. (**e**) Thermal expectation votes.

Figure 7 depicts the overall perceptual responses (thermal sensation votes, thermal comfort votes, humidity sensation votes, Borg’s rating of perceived exertion and thermal expectation votes.) under the four studied working conditions. At *I*_cl_ = 0.75 clo and WBGT = 15 °C, TSV gradually approached −1 during the time course, and most participants reported “cold” for TSVs. At WBGT = 28 °C, the mean overall TSVs with 0.37 and 0.75 clo inner garments were +1.16 and +1.43 (between “warm” and “hot”), respectively. No significant differences in TSV were found between the two types of inner garments. TSV exceeded +2 after 45 min, and the overall TSV was interpreted to be between “hot” and “very hot”. TSV represents the temperature sensations of the participants at a specific time point and does not reflect the actual body thermal status (heat accumulation/decay) of the participants. Thus, thermal comfort votes (TCVs) were also reported (Figure 7b). The discomfort felt by the participants increased as the duration of heat exposure and the level of heat stress increased. The mean TCVs with 0.75 clo inner garments were +0.85 (close to “slightly uncomfortable”) and +2.24 (between “uncomfortable” and “very uncomfortable”) at WBGTs of 15 °C and 28 °C, respectively. By contrast, TCVs with 0.37 clo inner garments were +1.2 (between “slightly uncomfortable” and “uncomfortable”) and +2.78 (close to “very uncomfortable”) at WBGTs of 28 °C and 32 °C, respectively. At WBGT = 28 °C, the discomfort level of participants with 0.75 clo inner garments was significantly higher than that with 0.37 clo inner garments. The TSV and TCV results were noticeably separated at 28 °C (Figure 7a,b) because the TSVs mainly focused on the physical sensations of a person, whereas the TCVs related to the psychological and physical sensations of a person. At WBGT = 28 °C, warm long-sleeved trousers were rarely worn in real life, unlike short-sleeved trousers. Therefore, under the joint effect of psychological and physical sensations and the human body dressing for 0.75 clo, the sense of psychological discomfort was enhanced, hence the clear separation.

As shown in Figure 7c, a humidity sensation vote (HSV) of 0 (“moderate”) with 0.75 clo inner garments is reported at WBGT = 15 °C. At WBGT = 28 °C, the HSV of participants rose to +1.06 (close to “slightly humid”). Similarly, HSVs rose to +1.51 with a strong sense of humidity at WBGT = 32 °C. When 0.37 clo inner garments were used, the HSVs of the participant were lower than those with 0.75 clo inner garments at WBGT = 28 °C. As shown in Figure 7d, Borg’s rating of perceived exertion (RPEs) is significantly different under different heat stress levels with the same inner garments. Given *I*_cl_ = 0.75 clo and WBGT = 15 °C, RPE was 12.20 (close to “relaxed”). At WBGT = 28 °C, the mean overall RPEs with 0.37 and 0.75 clo inner garments were 13.35 and 13.05 (“slightly tired”), respectively. RPEs rose to 15.31 (“tired”) at WBGT = 32 °C. At WBGT = 28 °C, the decrease in thermal resistance of the inner garments had little effect on the participants’ RPE. Figure 7e depicts the thermal expectation votes (TEV) of the participants. When *I*_cl_ = 0.75 clo and WBGT = 15 °C, the TEV of the participants gradually rose to +1 (“get warm”) as the experiment progressed, and 100% of the participants expected the temperature to warm up at 90 min. At WBGT = 28 °C, all the participants expected the temperature to cool down, and the participants with 0.75 clo inner garments expected the temperature to lower more than those with 0.37 clo inner garments. At WBGT = 32 °C, participants voted −1 (“get cool”) throughout the experiment.

### 3.3. Correlation between Physiological Parameters and WBGT (or Perceptual Responses)

**Figure 8:**
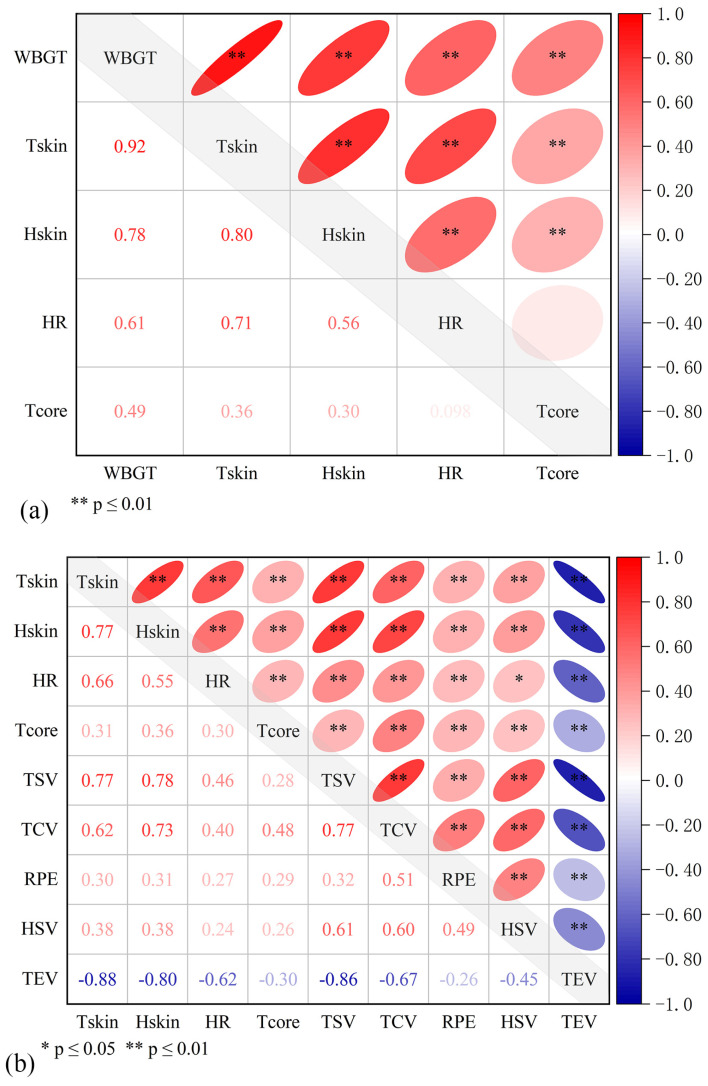
Correlation between physiological parameters and WBGT (or perceptual responses). (**a**) Correlation heatmap of physiological parameters and WBGT. (**b**) Pearson correlation analysis between subjective evaluation and physiological parameters.

A total of 342 groups of human physiological data reported in the current study were analyzed. The correlation heatmap is presented in Figure 8a. A significant positive correlation was found between *T*_skin_, *H*_skin_, HR, *T*_core_, and WBGT indexes at the *p* ≤ 0.01 level. The average skin temperature had the highest correlation coefficient (r = 0.92), indicating that compared with other indexes, *T*_skin_ was more significantly affected by environmental thermal stress.

A typical Pearson correlation analysis was performed on nine indicators of physiological parameters and perceptual responses (Figure 8b). At the *p* < 0.01 level, the results revealed significant positive correlations between *T*_skin_, *H*_skin_, *T*_core_, and TSV, TCV, and RPE. Physiological parameters, such as *T*_skin_ and *H*_skin_, and perceptual responses, such as TSV and TCV, exhibited the highest correlation. A significant positive correlation was determined between HR and HSV at the *p* < 0.05 level. Thermal expectation voting exhibited a significant negative correlation with other indicators at the *p* < 0.01 level.

### 3.4. Cooling Trials

#### 3.4.1. Physiological Responses

**Figure 9:**
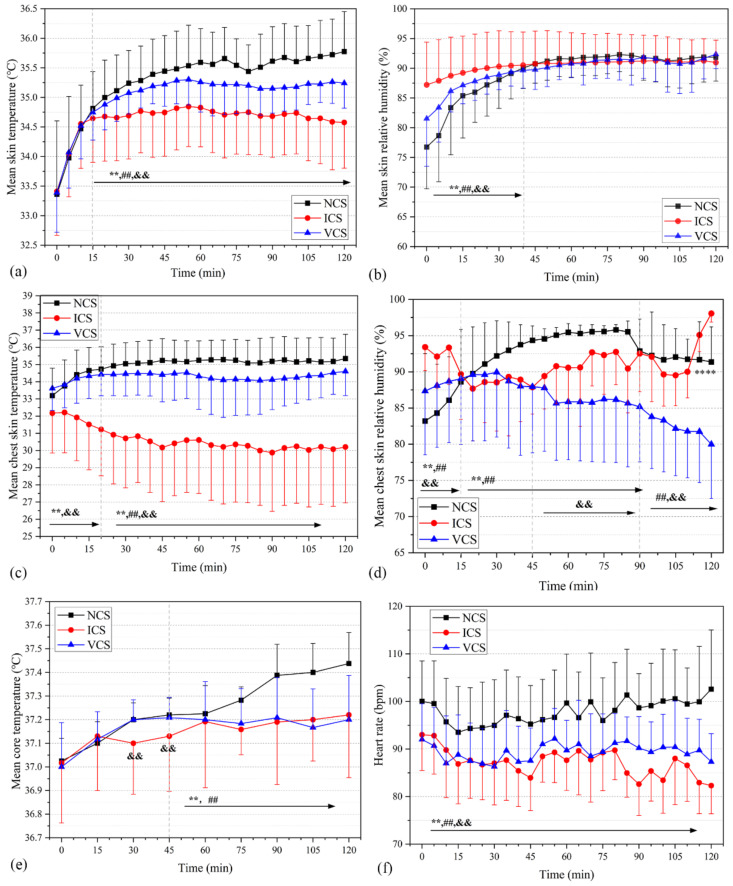
Temporal variations in physiological parameters in NCS, ICS, and VCS. (**a**) Mean skin temperature. (**b**) Mean skin relative humidity. (**c**) Mean chest skin temperature. (**d**) Mean chest skin relative humidity. (**e**) Mean core temperature. (**f**) Mean heart rate. **, *p* < 0.001 (NCS vs. ICS); ##, *p* < 0.001 (NCS vs. VCS); &&, *p* < 0.001 (ICS vs. VCS).

Active cooling measures at WBGT = 32 °C were observed by wearing innerwear with a thermal resistance of 0.37 clo or less. Figure 9a depicts the changes in mean skin temperature when two types of cooling clothing are used. During the first 15 min of the experiment, the mean skin temperature of each group increased rapidly by about 1 °C. No significant difference in *T*_skin_ was observed between NCS and ICS or NCS and VCS in the initial 15 min (*p*_1_ = 0.435, *p*_2_ = 0.452). After 15 min, the ICS group significantly suppressed the increase in mean skin temperature, and *T*_skin_ was well maintained at around 34.6 °C, with a slightly decreasing trend. Similarly, *T*_skin_ in VCS was significantly lower than that in NCS. Throughout the experiment, *T*_skin_ in the ICS and VCS groups decreased by 0.61 °C and 0.22 °C, respectively, relative to the NCS. *H*_skin_ in the NCS and VCS groups increased significantly in the first 40 min (Figure 9b), while *H*_skin_ in the ICS group remained constant, owing to the condensation of the water molecules in the air into water droplets when exposed to an ice-cold environment, resulting in increased skin humidity. After 40 min, no significant difference in *H*_skin_ was determined, remaining essentially constant at 91% under the three working conditions.

The ICS cooling suit is vest-shaped, causing the cooling effect to be concentrated in the upper torso and the air inlet of the VCS group to be located in the chest position. Thus, the temperature and humidity of the chest area were separately measured (Figure 9c). The findings were similar to the mean skin temperature, but the ICS group exerted a greater cooling effect on the chest. The highest degree of cooling occurred between the 5th and 45th min, gradually decreasing after 45 min; however, cooling remained significant in contrast to no cooling (i.e., NCS). The reason for this could be that the cooling effect weakened as the ice melted. The mean chest temperatures of the NCS, ICS, and VCS groups were 35.0 °C, 30.6 °C, and 34.3 °C, respectively, throughout the experiment. Figure 9d depicts the relative humidity responses in the chest area. The relative humidity levels were lower in the ICS and VCS groups than in the NCS group. The relative humidity variation in the ICS group more widely fluctuated and had a larger standard deviation, owing to the space between the ice bag cooling vest and the bodies of the participants because of differences in body size. The condensation of water vapor outside the ice bag contributed to the sudden increase in humidity at the 110th minute. Humidity in the VCS group gradually decreased because ventilation accelerated sweat evaporation and effectively reduced heat stress. Figure 9e presents the temporal variation in core temperature under the two cooling strategies and the no cooling method (i.e., NCS); *T*_core_ was significantly lower in ICS vs. VCS than in NCS at 30–120 min. No significant difference was found between NCS and ICS (*p* = 0.076) or NCS and VCS (*p* = 0.156). However, both cooling suits successfully prevented an increase in the mean core temperature of the participants. HRs in ICS and VCS were significantly lower than that of NCS (*p* < 0.001) (Figure 9f). Throughout the experiment, the HRs in ICS and VCS were reduced by 10.7 and 8.5 bpm, respectively, relative to that of NCS.

#### 3.4.2. Perceptual Responses

**Figure 10:**
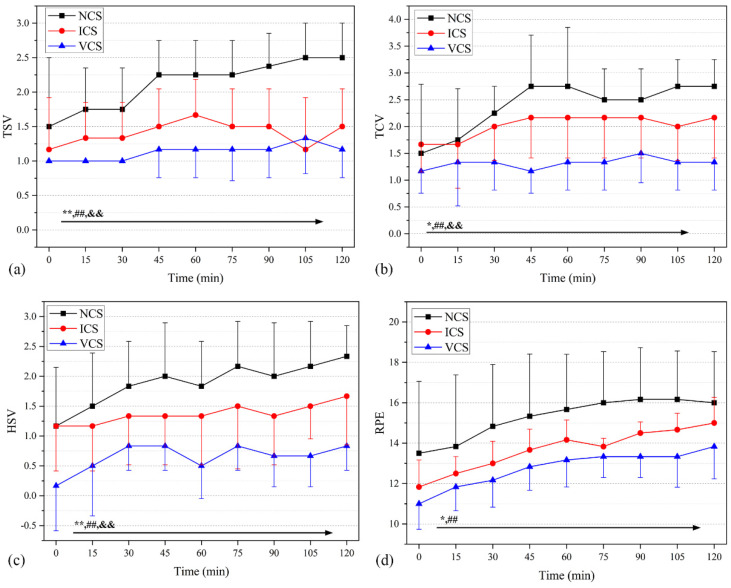
Comprehensive subjective evaluation voting in NCS, ICS, and VCS. (**a**) Thermal sensation votes. (**b**) Thermal comfort votes. (**c**) Humid sensation votes. (**d**) Borg’s rating of perceived exertion. *, *p* < 0.05, **, *p* < 0.001 (NCS vs. ICS); ##, *p* < 0.001 (NCS vs. VCS); &&, *p* < 0.001 (ICS vs. VCS).

Figure 10 presents the subjective evaluation parameters of the participants under the three cooling scenarios. Significant differences in TSVs were found between NCS and ICS (*p* = 0.001) and NCS and VCS (*p* < 0.001) throughout the experiment (Figure 10a). At the end of the experiment, TSVs in the NCS, ICS, and VCS groups were +2.50 (between “hot” and “very hot”), +1.50 (between “warm” and “hot”), and +1.17 (between “warm” and “hot”), respectively. TCVs in VCS were significantly lower than those in NCS throughout the experiment (*p* < 0.001), and TCVs in ICS were always lower than those in NCS throughout the 15–120th min (*p* = 0.047) (Figure 10b). At the end of the experiment, TCVs in the NCS, ICS, and VCS groups were +2.75 (close to “very uncomfortable”), +2.17 (between “uncomfortable” and “very uncomfortable “), and +1.34 (between “slightly uncomfortable” and “uncomfortable”), respectively.

NCS showed significantly higher HSVs than ICS and VCS within 120 min (Figure 10c). HSVs were significantly different under all three conditions (*p* < 0.001); at the end of the experiment, NCS, ICS, and VCS showed the highest HSV scores— (i.e., +2.34 (between “humid” and “very humid”), +1.67 (between “slightly humid” and “humid”), and +0.83 (between “neutral” and “slightly humid”), respectively). Figure 10d shows that the RPEs of ICS and VCS are clearly superior to that of NCV, indicating relatively low fatigue. No significant difference in RPEs was determined between ICS and VCS (*p* = 0.064). The RPE and NCS groups were 16 (tired), 15 (tired), and 13.83 (slightly tired) at 120 min, respectively.

## 4. Discussion and Limitations

In this study, the heat stress response of PPE-clad subjects was evaluated in three different environments (WBGTs = 15, 28, 32 °C) and at two thermal resistance levels of undergarments (*I*_cl_ = 0.37, 0.75 clo). The physiological parameters and perceptual responses are thus greatly affected by the environment, which is consistent with previous research results [21,31]. Local skin temperature is more significantly affected by environmental changes. When a participant sits in a low-temperature environment, vasoconstriction occurs in the skin, the blood circulation rate slows down, and heat transfer from the deep part of the body to the limps decreases. Particularly for the parts that are farther away from the heart and lack a thicker fat layer to remain warm, the more obvious the change in temperature [32]. Therefore, in a low-temperature environment with WBGT = 15 °C, the skin temperature of more prominent or slender parts, such as the scapula, arm, hand, leg, and so on are significantly affected. In a high-temperature environment, the skin temperature and relative humidity of the hand and head are relatively high. Numerous studies have also identified the negative effects of wearing PPE on the hands and the head [18,33], which has been verified in the current study.

In basic trials, participants could only maintain thermal comfort for a short period while seated with 0.75 clo inner garments at WBGT = 15 °C. During the extended time, they began to feel cold and desired more warmth. Additional inner clothing could easily reduce the cold sensation and assist participants in achieving thermal comfort. To achieve the desired thermal comfort, healthcare workers should wear higher-insulation inner garments when WBGT is 15 °C or lower. In the current study, wearing 0.75 clo inner garments made participants feel hot and uncomfortable when WBGT was raised to 28 °C. Despite using a set of lower-insulation inner garments (i.e., 0.37 clo, with a t-shirt and short pants), the participants were unable to maintain thermal comfort. This observation indicated that compensable heat stress occurred in such a mildly warm environment. Physiological and perceptual responses deteriorated even more when WBGT was raised to 32 °C with 0.37 clo inner garments. Previous studies have indicated that working in a high-temperature environment can increase the risk of heat stress and seriously affect the physical and mental health and work efficiency of personnel [34,35]. Accordingly, in the current study, when WBGT = 32 °C, personal cooling strategies should be applied to reduce uncompensable heat stress.

In the cooling trials, two sets of cooling suits were selected for natural air cooling and ice vest cooling. The use of ICS and NCS significantly reduced the heat stress experienced by the participants, and to a lesser extent, decreased heat stress in healthcare workers while working. However, carrying extra weight can significantly increase discomfort [36], particularly during long working hours. Consequently, ICS showed less improvement in perceptual response votes, compared with VCS. In addition, the current study only evaluated the cooling enhancement effect of currently available portable cooling devices on the market. ICS and VCS revealed numerous flaws during the experiment. Cadarette et al. [37] concluded that liquid cooling in the systemic circulation was more effective than cooling in the trunk alone. Dhandapani et al. [11] reported that 73.4% of people identified headaches as the adverse effects of wearing PPE, 91.7% complained of goggles getting fogged up, and 36.7% reported difficulty breathing. Human head and face discomfort was not well resolved [38,39]. VCS directly introduces filtered outdoor air, and the introduced air is not cooled down. Thus, in the current study, the improvement effect of VCS on human physiological parameters was not significant in the environment with WBGT = 32. Therefore, the air source cooling problem during ventilation cooling is worth considering [40], as is water vapor condensation during ice cooling [41]. The development of cooling suits is complex, and in future studies, further research needs to be conducted to achieve the enhanced cooling capacity for the thermal comfort of medical staff in high-temperature and -humidity environments to improve.

This study has some additional limitations that should be noted. First, the experimental sample size is small because field studies were greatly affected by outdoor weather conditions, and it was difficult to have experimental days that met the WBGT requirements. Second, the study findings may not accurately reflect the physiological and perceptual responses of professional healthcare workers or other age groups because only college students were recruited. Future research should include a broader range of populations. Third, variations in the fabrics and fitting methods used for the two kinds of inner garments selected in the experiment may affect the experimental results. Compared with 0.37 clo inner garments, the 0.75 clo inner garments showed higher moisture absorption. Fourth, in the human trials, the oral temperature was used to represent the core temperature. The limitation of the test apparatus for the oral temperature test is that it cannot be measured real-time, the measurement time interval is large, and the thermal state of the experimental object is affected during the experiment. In order to minimize this impact, the mask was not completely removed during the test, and the instrument was able to obtain data within 10 s. The participant would immediately wear the mask after the testing. Future studies should improve the measurement of the core temperature. The rectal or esophageal temperature should be reported [42,43]. Finally, future studies should include prolonged heat exposure to investigate the actual performance of various personal cooling strategies in transient-temperature and -humidity climates.

## 5. Conclusions

The effects of environmental heat stress, inner-layer insulation, and personal cooling strategy on the physiological and perceptual responses of PPE-clad young college students were investigated in this study. Three WBGT levels were chosen to represent cool, moderately warm, and warm conditions: 15 °C, 28 °C, and 32 °C, respectively. Two levels of inner-layer garments (0.37 clo and 0.75 clo) and two cooling strategies (natural air cooling and ice vest cooling) were considered. The following is a summary of key findings from this study:

(1) *T*_skin_ was 31.2 °C at WBGT = 15 °C with 0.75 clo inner garments. The low temperature environment significantly affected the skin temperature at the scapula, arm, hand, and leg. *H*_skin_ was maintained at 60–65% and HR was about 75.5 bpm, all of which were within the thermal comfort zone.
(2) At WBGT = 28 °C, *T*_skin_ plateaued at around 34.7 °C, and participants reported experiencing “hot” thermal sensations. Reducing the thermal resistance of the inner garment only slightly affected the participants’ physiological parameters, but the subjective evaluation index decreased as the thermal resistance of the inner garment was reduced. The insulation reduction of inner garments from 0.75 clo to 0.37 clo did not significantly improve the participants’ physiological thermal comfort.
(3) At WBGT = 32 °C, *T*_skin_ was maintained at 35.2–35.7 °C, *H*_skin_ was nearly 90% RH, *T*_core_ exceeded 37.1 °C, and the mean HR was 91.9 bpm. These results indicate that such a working scenario was uncompensable from the heat stress perspective. Hence, personal cooling to mitigate heat stress was required.
(4) Significant positive correlations were found between *T*_skin_, *H*_skin_, HR, *T*_core_, and WBGT values at the *p* ≤ 0.01 level, with *T*_skin_ exhibiting the highest correlation coefficient with WBGT (r = 0.92), indicating that *T*_skin_ was more significantly affected by the thermal stress of the external environment. *T*_skin_, *H*_skin_, HR, *T*_core_ were positively correlated with TSV, TCV, RPE (*p* < 0.01).
(5) When compared with that in NCS (no cooling), mean skin temperatures in ICS and VCS were reduced by 0.61 °C and 0.22 °C, respectively; chest temperature was as low as 30.6 °C; HRs were reduced by 10.7 and 8.5 bpm; and core temperatures were maintained at 37.2 °C with both personalized cooling strategies. *H*_skin_ in ICS was high because of moisture condensation from the ice packs. Perceptual responses in ICS and VCS improved significantly throughout the entire field trials, with VCS outperforming ICS in the individual cooling effect.

## Figures and Tables

**Table 1:** Details of instruments used in the study.

Parameters	Type	Range	Accuracy	Resolution
Air temperature	JI-IAQ-50	−20~120 °C	±0.3 °C	0.02 °C
Air humidity		0~100%	±2.5%	0.01%
Globe temperature		5~60 °C	±0.5 °C	0.1 °C
Air velocity		0.05~2 m/s	±0.03 m/s	0.01 m/s
Core temperature	YCT-2	32.0~42.9 °C	±0.1 °C	0.1 °C
Skin temperature	iButton	−20~85 °C	±0.5 °C	0.0625 °C
Skin humidity		0~100%	±5%	0.04%
Heart rate	POLAR	30~240 bpm	±1 bpm	1 bpm

**Table 2:** Anthropometric information of participants.

Gender	Age	Height (cm)	Weight (kg)	Body Mass Index	Body Surface Area (m^2^)
Males	22.0 ± 0.6	177.2 ± 4.3	68.6 ± 4.6	21.8 ± 1.6	1.93 ± 0.17
Females	21.9 ± 0.7	163.5 ± 5.5	56.2 ± 12.3	21.0 ± 3.3	1.69 ± 0.07
Overall	21.9 ± 0.6	170.0 ± 8.5	61.7 ± 10.1	21.3 ± 2.4	1.80 ± 0.17

**Table 3:** Perceptual response voting scales.

Thermal Sensation Vote	Thermal Comfort Vote	Borg’s Rating of Perceived Exertion	Humid Sensation Vote	Thermal Expectation
+3 very hot +2 hot +1 warm 0 neutral −1 cool −2 cold −3 very cold	0 comfortable 1 slightly uncomfortable 2 uncomfortable 3 very uncomfortable 4 intolerable	6 effortless 7~8 extremely easy 9~10 very easy 11~12 easy 13~14 slightly tired 15~16 tired 17~18 very tired 19 extremely tired 20 exhausted	+3 very humid +2 humid +1 slightly humid 0 neutral −1 slightly dry −2 dry −3 very dry	+1 get warm 0 neutral −1 get cool

**Table 4:** Experimental PPE and ensembles.

	Type	Clothing Composition	Clothing Display
Basic trial	Dress 1 *I*_cl1_ = 0.75 clo	PPE + Trousers + Long sleeve top + Short sports socks + Sneakers	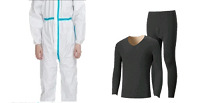
	Dress 2 *I*_cl2_ = 0.37 clo	PPE + Short trousers + T-shirt + Short sports socks + Sneakers	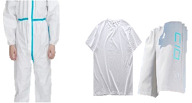
Cooling trial	ICS	PPE + Dress2 + Ice bag cooling vest	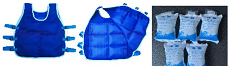
	VCS	PPE+ Dress2 + Ventilation cooling system	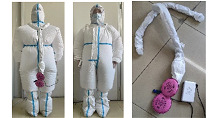

## Data Availability

Not applicable.

## Nomenclature

*H*_skin_	mean skin relative humidity, %
*I*_cl_	thermal resistance of clothing, clo
*RH*	relative humidity, %
*t*_a_	dry bulb temperature, °C
*T*_core_	mean core temperature, °C
*t*_g_	black globe temperature, °C
*T*_skin_	mean skin temperature, °C
*t*_w_	wet bulb temperature, °C
*v*	air velocity, m/s
Abbreviation	
COVID-19	Coronavirus Disease-2019
HR	heart rate, bpm
HSV	humid sensation votes
ICS	ice bag cooling system
NCS	no cooling system
RPE	Borg’s rating of perceived exertion
TCV	thermal comfort votes
TEV	thermal expectations votes
TSV	thermal sensation votes
VCS	ventilation cooling system
WBGT	wet bulb globe temperature, °C
Subscripts	
cl	a set of clothing
i	single piece clothes
